# Anti-Osteoclast Effect of Exportin-1 Inhibitor Eltanexor on Osteoporosis Depends on Nuclear Accumulation of IκBα–NF-κB p65 Complex

**DOI:** 10.3389/fphar.2022.896108

**Published:** 2022-08-30

**Authors:** Junchun Chen, Dezhi Song, Yang Xu, Liwei Wu, Lili Tang, YuanGang Su, Xiaoxiao Xie, Jinmin Zhao, Jiake Xu, Qian Liu

**Affiliations:** ^1^ Research Centre for Regenerative Medicine, Orthopaedic Department, The First Affiliated Hospital of Guangxi Medical University, Nanning, China; ^2^ Collaborative Innovation Centre of Regenerative Medicine and Medical BioResource Development and Application Co-Constructed by the Province and Ministry, Guangxi Medical University, Nanning, China; ^3^ Guangxi Key Laboratory of Regenerative Medicine, Guangxi Medical University, Nanning, China; ^4^ School of Biomedical Sciences, The University of Western Australia, Perth, WA, Australia

**Keywords:** eltanexor (KPT-8602), selinexor (KPT-330), NF-κB, XPO1, osteoporosis

## Abstract

Osteoporosis affects around 200 million people globally, with menopausal women accounting for the bulk of cases. In the occurrence and development of osteoporosis, a key role is played by osteoclasts. Excessive osteoclast-mediated bone resorption activity reduces bone mass and increases bone fragility, resulting in osteoporosis. Thus, considerable demand exists for designing effective osteoporosis treatments based on targeting osteoclasts. Eltanexor (Elt; KPT-8602) is a selective nuclear-export inhibitor that covalently binds to and blocks the function of the nuclear-export protein exportin-1 (XPO1), which controls the nucleus-to-cytoplasm transfer of certain critical proteins related to growth regulation and tumor suppression, such as p53, IκBα [nuclear factor-κB (NF-κB) inhibitor *α*] and FOXO1; among these proteins, IκBα, a critical component of the NF-κB signaling pathway that primarily governs NF-κB activation and transcription. How Elt treatment affects osteoclasts remains poorly elucidated. Elt inhibited the growth and activity of RANKL-induced osteoclasts *in vitro* in a dose-dependent manner, and Elt exerted no cell-killing effect within the effective inhibitory concentration. Mechanistically, Elt was found to trap IκBα in the nucleus and thus protect IκBα from proteasome degradation, which resulted in the blocking of the translocation of IκBα and NF-κB p65 and the consequent inhibition of NF-κB activity. The suppression of NF-κB activity, in turn, inhibited the activity of two transcription factors (NFATc1 and c-Fos) essential for osteoclast formation and led to the downregulation of genes and proteins related to bone resorption. Our study thus provides a newly identified mechanism for targeting in the treatment of osteoporosis.

## Introduction

Osteoporosis is a systemic bone disease characterized by bone mass loss, damage to bone tissue architecture, and increased bone fragility, all of which increase the risk of fractures (Wu et al., 2018). With the world’s population aging, osteoporosis has arisen as a frequent illness that endangers human health and safety (Wang Y. et al., 2020). According to the latest epidemiological survey and research, osteoporosis affects >200 million people worldwide, with women aged >50 years old accounting for >40% of the osteoporosis cases, and bone fractures occur in most of the patients (Zhu et al., 2019; Arandjelovic et al., 2021). Adult bone homeostasis is dependent on a coordinated bone-remodeling process that comprises osteoclast-mediated bone resorption and osteoblast-mediated bone synthesis (Zheng et al., 2020). Osteoporosis develops when the body’s bone metabolism becomes unbalanced, specifically when the amount of bone resorption exceeds the amount of bone production (Rached et al., 2010). When osteoclasts are overactivated, the bone-metabolism balance is disrupted, and this disruption can then lead to various bone lytic diseases such as osteoporosis and rheumatoid arthritis (Ahn et al., 2018; Minoshima et al., 2019). Accordingly, inhibition of osteoclast activity and function has long been regarded a significant target in the treatment of osteoporosis (Kim et al., 2020), and thus, investigating targeted blockade of intracellular signal transduction in osteoclasts for osteoporosis treatment holds considerable clinical significance.

Osteoclasts are multinucleated cells formed by the fusion of macrophage progenitor cells (monocytes). The production of mature osteoclasts is regulated by multiple factors such as genetics, internal environment, and external physical factors, and among the factors involved, two cytokines macrophage colony-stimulating factor (M-CSF) and receptor activator of nuclear factor-κB (NF-κB) ligand perform important regulatory roles (RANKL) (Park-Min et al., 2014). M-CSF is secreted by osteoblasts and mesenchymal cells, and activates the extracellular signal-regulated kinase 1/2 (ERK1/2) and phosphatidylinositol 3-kinase/protein kinase B (PI3K/AKT) pathways by binding to the receptor tyrosine kinase c-fms, which regulates osteoclast precursor cell proliferation and survival (Kim et al., 2017). RANKL is secreted by osteoblasts, T cells, and endothelial cells and binds to the receptor activator of NF-κB (RANK) on the surface of osteoclast precursor cells to recruit tumor necrosis factor (TNF) receptor-associated factor 6 (TRAF6) for further activation of signaling pathways such as the NF-κB, mitogen-activated protein kinase (MAPK), and calcium-shock pathways. Activation of these pathways can enhance the expression of tartrate-resistant acid phosphatase (TRAcP/Acp5), cathepsin K (CTSK), V-ATPase d2 (ATP6V0D2), integrin αV, and other osteoclast-related genes and proteins (Kim et al., 2017; Lee et al., 2020). These intertwined and complex signaling pathway systems suggest a basis and direction for targeted therapy of osteoporosis in the clinic and also provide a reference for the prevention of osteoporosis; however, additional investigations must be conducted to clearly demonstrate or supplement what is known regarding the underlying mechanisms.

NF-κB is a transcription factor that governs cell proliferation and differentiation, and the NF-κB signaling pathway regulates the body’s inflammatory, immunological, and other stress responses (Roccaro et al., 2008; Ke et al., 2015). In the classic NF-κB signaling pathway, NF-κB associates with NF-κB inhibitor *α* (IκBα) to form a complex that shuttles between the cytoplasm and the nucleus; this shuttling occurs through nuclear localization signal (NLS)-dependent nuclear import and nuclear-export protein-1 (exportin-1/XPO1)-dependent nuclear export (Lang and Jou, 2021). Exogenous stimulating factors such as RANKL, TNF-α, and interleukin (IL)-1 activate the IκB kinase (IKK) complex in osteoclasts, following which IKK phosphorylates IκB and thus causes IκB degradation and the release of NF-κB; the released NF-κB subsequently translocates to the nucleus, where it binds to particular DNA sequences, regulating the transcription of genes involved in this process, including genes responsible for inducing osteoclast differentiation (Schuman et al., 2009). Another activation route, termed as the alternative pathway, includes inducible proteolytic degradation of the NF-κB2/p100 REL protein. The activation of NF-κB-inducing kinase (NIK) and IKKa results in the phosphorylation of p100 and the proteasome processing of p100 to p52. This process primarily creates p52/RELB heterodimers, which also translocate into the nucleus and control a variety of target genes (Xu et al., 2009). Therefore, inhibiting the activation of the NF-κB signaling pathway is one of the effective methods to regulate osteoclast differentiation. The regulatory methods used in previous studies have focused on inhibiting the activation of IKK, particularly by targeting the phosphorylation and nuclear translocation of IκBα, and thereby suppressing RANKL-induced activation of the NF-κB pathway (Takada et al., 2005). Here, we sought to block signal transduction at the level of subcellular localization, and further definition of the mechanisms involved in subcellular localization of latent NF-κB complexes would not only expand our understanding of NF-κB regulation, which is expected to provide a new avenue for inhibiting the activation of the NF-κB pathway. More importantly, this approach is likely to provide a new class of drug targets to attenuate NF-κB functions selectively.

The nuclear-export receptor protein XPO1, also known as chromosomal region maintenance 1 (CRM1), is widely expressed (Lundberg et al., 2016). XPO1 transports a variety of tumor-suppressor and regulatory proteins, including p53, p27, and IκB, which are examples of cargo proteins carried from the nucleus to the cytoplasm *via* the nuclear pore (Alexander et al., 2016). Eltanexor (Elt), a small-molecule inhibitor of XPO1, produces antitumor effects in leukemia, multiple myeloma, and lymphoma (Taylor-Kashton et al., 2016), and the first-generation inhibitor Sel has been shown to specifically inhibit XPO1-mediated nuclear protein export and to inhibit the NF-κB signaling pathway by increasing the nuclear accumulation of IκBα (Nair et al., 2017). The nuclear translocation and accumulation of IκBα is a critical mechanism for regulating NF-κB-dependent transcription of proinflammatory and antiapoptotic genes (Finkin-Groner et al., 2015). However, the effect of Elt-mediated XPO1 inhibition of the NF-κB signaling pathway in osteoclasts remains unclear. Considering the crucial function of NF-κB in the growth and differentiation of osteoclasts, we investigated whether Elt could play a potential role in suppressing osteoclasts.

There are currently several medications available to treat osteoporosis. Despite the fact that natural chemicals and plant extracts have been endlessly developed, the creation of more effective medications is always stalled due to their more or less harmful and adverse effects (Kim et al., 2021). XPO1 protein inhibitors are medicines that are covalently bonded (Sweet et al., 2020). Covalently bound medications have some distinct benefits over traditional drugs, such as high affinity and potency, specific selectivity, long binding duration, and superior pharmacokinetic quality. The key clinical molecule is selinexor (Sel; KPT-330), a first-generation XPO1 inhibitor. Sel has been investigated in phase I and II clinical trials for a variety of solid and hematological cancers (Subhash et al., 2018; Laubach et al., 2019). Elt, a second-generation XPO1 inhibitor, has fewer preclinical and clinical studies than Sel, but has been shown to be a more potent XPO1 inhibitor with the same efficacy as Sel (Vercruysse et al., 2017). Their role in osteoporosis against osteoclasts is unknown. Initially, we evaluated the cytotoxic effects of Elt and Sel, two generations of small-molecule XPO1 inhibitors, on bone marrow-derived macrophages (BMMs) and the inhibitors’ impact on osteoclast development *in vitro*. Next, we selected the second-generation inhibitor Elt, which exhibited less toxicity but a stronger inhibitory effect than the first-generation inhibitor Sel, for further analysis and investigated the mechanism of action of Elt. Lastly, we established mouse osteoporosis model, the ovariectomized (OVX) mouse, by using bilateral oophorectomy and evaluated the protective effect of Elt on bone loss.

## Methods

### Reagents and Antibodies

Sel and Elt (both >99% pure) were purchased from APE × BIO (Shanghai Weihuan Biotechnology Co., Ltd.; Cas No. 1642300-52-4, Cas No. 1393477-72-9 respectively). Dimethyl sulfoxide (DMSO) was purchased from Sigma-Aldrich (St. Louis, MO, United States), and Elt was dissolved in 40 mM DMSO as a storage solution; subsequently, an appropriate amount of the inhibitor was diluted in phosphate-buffered saline (PBS) or α-modified minimal essential medium (α-MEM) containing 10% fetal bovine serum (FBS), 1% penicillin/streptomycin (P/S), and 50 ng/ml M-CSF to the working concentration for use *in vitro* and *in vivo*. α-MEM, P/S solution, and Gibco was the source of FBS (Thermo Fisher Scientific, Waltham, MA, United States). R&D Systems provided recombinant mouse M-CSF and recombinant mouse RANKL (Minneapolis, MN, United States). Primary antibodies (mouse and rabbit) against the following protein targets were from commercial sources: phosphorylated (p)-ERK, ERK, p-JNK (c-Jun N-terminal kinase), JNK, p-p38, p38, p-p65, p65, IKKα, IKKβ, β-actin, c-Fos, integrin αV, and V-ATPase d2, Cell Signaling Technology (Boston, MA, United States); IκBα and NFATc1, Santa Cruz Biotechnology (Santa Cruz, CA, United States); CTSK, Abcam (Cambridge, United Kingdom); and lamin B1, Proteintech (Rosemont, IL, United States).

### Cell Separation and Culture

Specific pathogen-free (SPF) 6-week-old female C57BL/6j mice were obtained and raised at Guangxi Medical University’s Experimental Animal Center (Nanning, Guangxi, China) to obtain BMMs in the femur and tibia, with the consent of the Animal Ethics Committee of Guangxi Medical University. Cell isolation procedures were: use a 1 ml syringe to suck the culture media and cleanse the separated femur and tibia bone marrow cavities in a biological safety cabinet, repeat 2–3 times to acquire additional bone marrow-derived macrophages, collect the cell suspension, and filter with a 70 μm filter tissue and cell clumps in excess. Then centrifuge for 3 min at 1,500 rpm, remove the supernatant, and resuspend the cells in -MEM complete media with 50 ng/ml M-CSF, 10% FBS, and 1% P/S, and transfer to a T75 culture flask. Incubate the cells at 37°C with 5% CO_2_, replace the media every other day, and utilize adherent cells for following studies.

BMMs were seeded into 96-well plates at a density of 6 × 10^3^ cells per well and cultured overnight until totally adherent. The next day, BMMs were stimulated with RANKL (50 ng/ml) alone or in combination with Elt (0, 25, 50, 75, and 100 nM). Every other day, the medium was replaced, and additional RANKL-stimulated BMMs were introduced to induce osteoclast production until the appearance of osteoclasts on the sixth day. BMMs without RANKL stimulation served as the negative control, while BMMs without RANKL stimulation served as the comparative pair. For more than 30 min, the cells were fixed in a 4% paraformaldehyde (PFA) solution at room temperature. Stained with TRAcP (Sigma-Aldrich), an excellent marker of osteoclast activity, and whole hole pictures were captured using Cytation 5 (BioTek Instruments Inc., Winooski, VT, United States). TRAcP-positive multinucleated cells with more than three nuclei were identified as osteoclasts, and the impact on preventing osteoclast development was measured by counting.

### Cell Viability and Proliferation Assay

Med Chem Express provided the Cell Counting Kit-8 (CCK-8) for this study. (Monmouth, NJ, United States). BMMs were seeded into 96-well plates at 8 × 10^3^ cells/well, cultured overnight until completely adhered, and treated on the next day with or without Sel or Elt (0, 25, 50, 75, 100, and 125 nM) for 48 h; following that, each well received a CCK-8 solution, and the cells were grown in the dark for 2 h. Lastly, the absorbance at 450 nm of the liquid in the wells was measured using a Tristar2 LB 942 multipurpose microplate reader (Berthold Technologies Gmbh & Co., KG, Baden-Württemberg, Germany).

### Actin Belt Staining

Osteoclasts were cultured as described in the preceding subsection, the cells were fixed in 4% paraformaldehyde (PFA), then gently rinsed three times with PBS, permeabilized for 5 min at room temperature with 0.1% Triton X-100 (Beijing Solarbio Science & Technology, Beijing, China), then blocked for 30 min in PBS with 3% BSA, and stained for 45 min with rhodamine-conjugated phalloidin (Yeasen Bioteck, Shanghai, China) (dissolved in 0.2% BSA-PBS). Following that, PBS was used to rinse the cells and DAPI (Santa Cruz, CA, United States) (in 0.2% BSA-PBS) was used to stain them for 5 min before being washed with PBS and then with BioTek, and photographed using RFP (red fluorescence protein) and DAPI fluorescence channels under a microscope.

### Bone-Resorption Activity Analysis

Zhejiang Zhuoteng Biotechnology Co., Ltd. supplied the bovine bone slices (Zhejiang, China). The slices were marked with a pencil in a sterile environment and gently shaken in 75% alcohol for 48 h to ensure complete sterilization, and then transferred into PBS and shaken for another 48 h to replace the residual alcohol. Next, the slices were shaken in α-MEM complete medium for >48 h and then washed with the PBS to achieve effective cell adhesion and lastly, transferred into 96-well plates that were placed in a biological safety cabinet in which the plates were treated with ultraviolet radiation, and the samples were dried. BMMs were inoculated into 6-well plates at 2 × 10^5^ cells/well, allowed to adhere to the walls overnight, and cultured with α-MEM complete medium (containing 50 ng/ml each of RANKL and M-CSF) until small osteoclasts started to form (confirmed by viewing under a microscope). The small osteoclasts being formed were collected and inoculated at 1× 10^4^ cells/well on the pretreated bovine bone slices, which were then placed in an examination set up featuring a controllable observation aperture. The cells were treated with Elt (0, 50, and 100 nM) the next day, and 2 days later, mature osteoclasts were examined through the observation aperture. At this stage, the cells in the control wells were subjected to TRAcP staining in order to count multinucleated cells. The cells were removed from the bovine bone slices before they were cleaned and treated with a particular fixing solution for analysis under an electron microscope as reported (Wang Q. et al., 2020). The electron microscope used was a Regulus 8100 (Hitachi, TYO, Japan). The typical absorption area was photographed and then analyzed using ImageJ software (NIH, Bethesda, MD, United States) to calculate the percentage of the area absorbed by osteoclasts. ImageJ software was used to calculate the bone-resorption area.

### Reverse-Transcription and Real-Time PCR

BMMs were seeded at 1.2 × 10^4^ cells/well in 6-well plates and allowed to adhere overnight before being treated with Elt (0, 50, 100 nM) in -MEM complete media containing 50 ng/ml RANKL and M-CSF and cultrued until osteoclast formation was visible under a microscope. The cells were next lysed on ice by adding TRIzol (Thermo Fisher Scientific), and total RNA was extracted using a Total RNA Extraction Kit and then reverse-transcribed and amplified (RevertAid First Strand cDNA Synthesis Kit, Thermo Scientific™). Total RNA concentration was measured using a NanoDrop™ One/One^C^ ultra-micro UV spectrophotometer (Thermo Scientific™), and lastly, real-time quantitative PCR (qPCR) was performed on a LightCycler®96 system (Roche, Basel, Switzerland). PCR amplification was performed using these conditions: denaturation at 95°C for 10 min, followed by 55 cycles at 95°C for 15 s, 60°C for 15 s, and 72°C for 40 s. The primer sequences used for osteoclast-related genes are listed in Table 1. The target genes were compared with the internal-reference gene *Gapdh* (which encodes glyceraldehyde 3-phosphate dehydrogenase), and the positive-control wells were used for standardization and statistical analysis.

**TABLE 1:** Primer sequences for qPCR.

Gene	Forward (5′-3′)	Reverse (5′-3′)
*Nfatc1*	GGT​GCT​GTC​TGG​CCA​TAA​CT	GAA​ACG​CTG​GTA​CTG​GCT​TC
*c-Fos*	CCA​GTC​AAG​AGC​ATC​AGC​AA	AAG​TAG​TGC​AGC​CCG​GAG​TA
*Ctsk*	AGG​CGG​CTC​TAT​ATG​ACC​ACT​G	TCT​TCA​GGG​CTT​TCT​CGT​TC
*Mmp9*	GAA​GGC​AAA​CCC​TGT​GTG​TGT​T	AGA​GTA​CTG​CTT​GCC​CAG​GA
*Dcstamp*	TCT​GCT​GTA​TCG​GCT​CAT​CTC	ACT​CCT​TGG​GTT​CCT​TGC​TT
*Acp5*	CAC​TCC​CAC​CCT​GAG​ATT​TGT	CAT​CGT​CTG​CAC​GGT​TCT​G
*Gapdh*	AAC​TTT​GGC​ATT​GTG​GAA​GG	ACA​CAT​TGG​GGG​TAG​GAA​CA

### Western Blotting

To analyze the early and late effects of Elt on the signaling pathways related to osteoclast activation, BMMs were inoculated into 6-well plates at 6 × 10^5^ or 1 × 10^5^ cells/well, and then, control and drug-treatment groups were generated; the plates were placed in an incubator overnight to allow cells to adhere to the plate walls. The early and late effects of Elt were investigated thus: the cells in the control and drug-treatment groups were starved through incubation (in parallel) in serum-free medium for 3 h, after which the cells were treated with or without Elt (100 nM) for 0, 5, 10, 20, 30, or 60 min or for 0, 1, 3, and 5 days while concurrently stimulating the cells in the two groups with 50 ng/ml RANKL. Before being lysed using RIPA buffer (APE×BIO) containing protease inhibitors, the cells were rinsed with PBS, PMSF (Beijing Solarbio Science & Technology), and phosphatase inhibitors (Beijing ComWin Biotech, Beijing, China), and then, the extracted proteins were collected through centrifugation. After mixing with a protein-loading buffer, the proteins were separated using 10% SDS-PAGE and transferred to nitrocellulose membranes (Thermo Fisher Scientific, Shanghai, China), which were blocked in TBST with 5% skimmed milk powder for 1 h at room temperature. After that, the membranes were incubated in primary antibody solutions overnight at 4°C with moderate shaking, followed by 1 h in the dark with appropriate secondary antibodies diluted in a 5% skimmed milk powder solution. Lastly, an ImageQuant LAS-4000 system (GE Healthcare, Chicago, IL, United States) was used for scanning the membranes, and the scanned images were statistically and quantitatively analyzed using ImageJ software.

### Nuclear and Cytoplasmic Extraction

BMMs were inoculated into 6-well plates at 1 × 10^6^ cells/well, and the plates were placed in an incubator overnight to allow the cells to adhere to the walls. After establishing negative-control, positive-control, and drug-treatment groups, the drug-treatment and positive-control groups were treated with or without Elt (100 nM) for 24 h and concurrently stimulated with 100 ng/ml RANKL. The cytoplasmic and nuclear proteins were separated as indicated by the manufacturer using a NE-PER Nuclear and Cytoplasmic Extraction Kit (ThermoScich #78833). Western blotting was used to examine cytoplasmic and nuclear proteins, with the extraction efficiency being evaluated based on the levels of β-actin (marker for cytoplasm) and lamin B1 (marker for nucleus).

### OVX Mouse Model

C57BL/6J female mice (10 weeks old, 18.2 ± 1.1 g) were acquired from Changsha Tianqin Biotechnology Co., Ltd. (Changsha, China) and bred in Guangxi Medical University’s SPF mouse breeding laboratory. The mice were brought to our laboratory and allowed to adapt to the new environment for 1 week before experiments. The Animal Experiment Ethics Committee of Guangxi Medical University (Nanning, China) approved the animal study. In the study, OVX + vehicle group, OVX + 100 ng/kg estrogen E2 group, OVX + 0.075 mg/kg Elt group, and OVX + 0.15 mg/kg Elt group were randomly assigned to 40 mice (*n* = 8 each). The mice were sedated with an intraperitoneal injection of tribromoethanol solution (15 mg/kg), and in the case of the OVX group, bilateral ovaries were removed from the dorsal approach. For the mice in the sham operation group, the abdominal cavity was opened, and the ovaries were visualized but not removed before surgically closing the abdominal cavity. The mice in the Elt groups were injected intraperitoneally with Elt every 2 days beginning 1 week after surgery, the mice in the OVX + E2 group were injected intraperitoneally with E2, and the mice in the sham operation group and the OVX + vehicle group were injected intraperitoneally with the same but with 0.9% saline in 0.1% DMSO starting 1 week after surgery. All of the mice’s right femurs were extracted and studied with a high-resolution micro-computed tomography (micro-CT) scanner (SkyScan 1176; Bruker, Kontich, Belgium) as reported (Yang et al., 2021). The left femurs were isolated for histological or immunohistochemical analysis.

### Micro-CT and Histological Analyses

Isolated mouse bilateral femurs (see subsection on OVX model) were placed in 4% PFA for 48 h, after which the right femurs were transferred to a 75% alcohol fixation solution for subsequent micro-CT scanning. The main parameters used for the femur scanning were as follows: 65 kV, 382 μA, and 9.0 µm scan interval. After scanning, sections of interest (0.9 mm thick) were chosen for 3D reconstruction, and the data was processed with Mimics 19.0, Magics 19.01, and ABA special bone-analysis software. The number of trabeculae (Tb.N), bone volume/tissue volume (BV/TV), trabecular thickness (Tb.Th), and trabecular separation (Tb.S) were the outcome measures (Tb.Sp). After being fixed in 75% alcohol for 24 h, the left femurs were decalcified in a 10% EDTA solution at 37°C for 20 days, and then the samples were embedded in paraffin, sectioned at a thickness of 5 μm, and deparaffinized before staining. Hematoxylin and eosin (H&E) staining and TRAcP staining were applied to the femoral slices, which were subsequently scanned and evaluated using an optical microscope and imaging equipment (EVOS FL automatic cell-imaging system) as reported (Tian et al., 2020). Bioquant Osteo software (Nashville, TN, United States) was used for statistical analysis of OC on each bone surface.

### Statistical Analysis

All of the experiments were done at least three times independently, and the findings are shown as mean standard deviation (SD). For statistical analysis and drawing quantitative statistics, Prism 8 (GraphPad Software, Inc., San Diego, CA, United States) was used. To evaluate differences between more than two groups, one-way ANOVA plus Tukey’s test or Kruskal Wallis analysis (nonparametric ANOVA) plus Dunn’s multiple comparison was employed. The effects of time and different treatment groups were investigated using a two-way ANOVA. A *p* value of less than 0.05 (*p* < 0.05) was used to determine statistical significance.

## Results

### Sel and Elt Inhibit RANKL-Induced Osteoclast Formation *in vitro*

**FIGURE 1:**
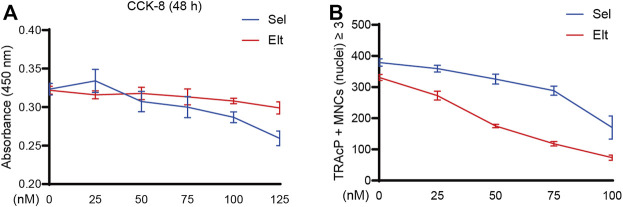
The effects of Sel and Elt on cell cytotoxicity and osteoclast inhibition *in vitro*. **(A)** Cell viability of Sel and Elt-treated BMMs at 48 h. **(B)** Trend of Sel and Elt on RANKL-induced osteoclast inhibition. The above data are expressed as the mean ± SD; *n* ≥ 3. Differences arise from inside groups.

**FIGURE 2:**
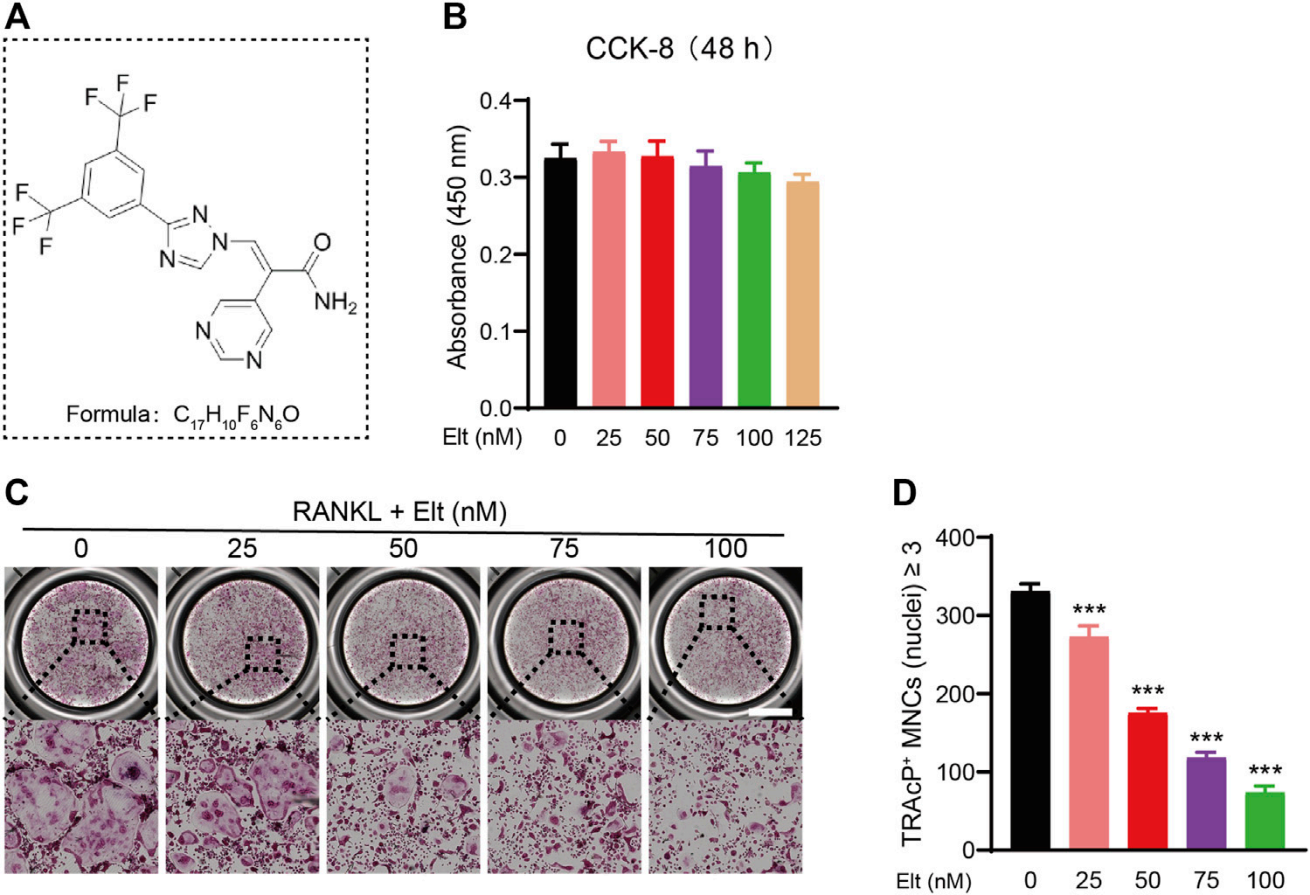
Elt inhibits RANKL-induced osteoclast formation *in vitro*. **(A)** The chemical structure and formula of and Elt. **(B)** Cell viability of Elt-treated BMMs at 48 h. **(C)** BMMs were stimulated with RANKL for 5 days, and representative images of TRAcP staining showed that Elt inhibited osteoclast formation in a dose-dependent manner (scale = 1,000 μM). **(D)** Quantification of the number of TRAcP^+^ multinucleated cells (nuclei ≥ 3). The above data are expressed as the mean ± SD; *n* ≥ 3; *p < 0.05 compared to control group.

The potential cytotoxicity of Sel and Elt toward BMMs was evaluated using the CCK-8 assay. At the concentrations tested in this study, Elt did not overtly affect BMM survival, but Sel showed an effect on BMM survival at higher concentrations (Figure 1A). Meanwhile, at the same dose, Elt had a stronger inhibitory effect on osteoclasts than Sel (Figure 1B). Therefore, we next investigated the effect of Elt on the differentiation of BMMs into osteoclasts *in vitro*: RANKL induced the differentiation of osteoclast precursors in the control group (no Elt treatment) into TRAcP-positive multinucleated mature osteoclasts; however, in the groups treated with Elt at 25–100 nM, we observed a dose-dependent inhibition of the size and number of mature osteoclasts than Elt-treated (Figures 2A–D). These results confirmed the safety of Elt and provided a basis for selecting the effective drug concentration.

### Elt Attenuates Formation of F-Actin Bands and Bone-Resorption Activity of Osteoclasts

**FIGURE 3:**
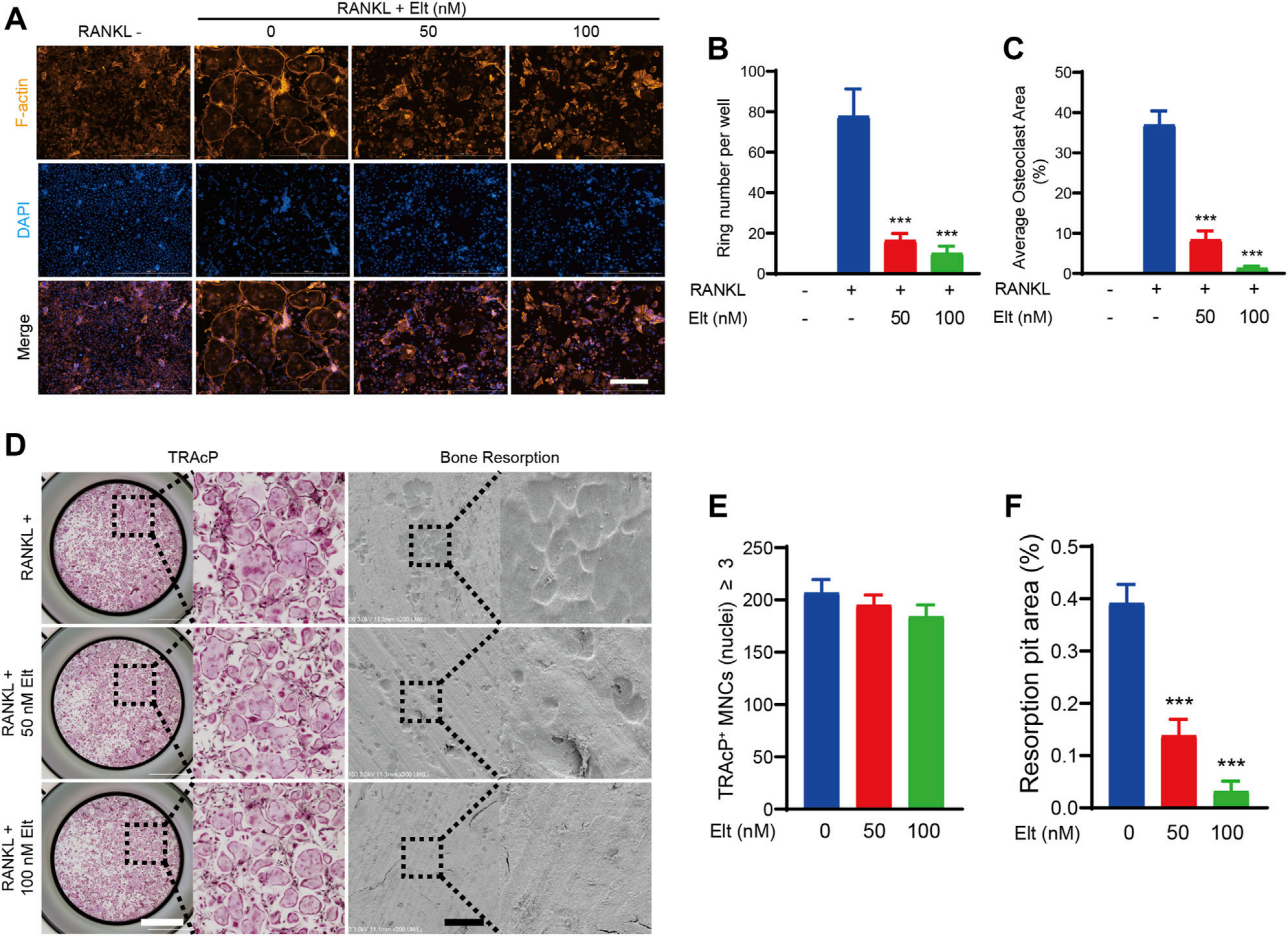
Elt attenuates formation of F-actin belts and bone-resorption activity of osteoclasts. **(A)** Representative images of F-actin belts formation in osteoclasts treated with different concentrations of Elt. The actin cytoskeleton (Orange red) and nucleus (blue) of osteoclasts were stained (scale = 500 μM). **(B,C)** Quantification of the belts number per well (*n* = 3 per group). **(D)** Representative images of TRAcP staining cells (scale = 1,000 μM) and bone resorption pits (scale = 500 μM). **(E,F)** Quantitative analysis of the number of TRAcP^+^ cells per well (96-well plate) and resorbed area on bone slices. The above data are expressed as the mean ± SD; *n* = 3; *p < 0.05 relative to RANKL-induced controls.

The bone-resorption activity of mature osteoclasts starts from the “sealing zone” formed between the cell membrane and the attached bone surface. The hallmark cytoskeletal structure of this area is the F-actin ring formed in the multinucleated mature osteoclasts (Kim et al., 2017). We studied the cytoskeleton of mature osteoclasts and estimated the number of nuclei in single cells using rhodamine-phalloidin and DAPI labeling, respectively, to evaluate the influence of Elt on cell morphological alterations associated to bone-resorption activity of mature osteoclasts. Treatment of osteoclasts with 50 and 100 nM Elt significantly reduced the number of F-actin belts and the average number of nuclei per cell (Figures 3A–C). Furthermore, to analyze the effect of Elt treatment on the bone-resorption activity of osteoclasts, we inoculated osteoclasts that had undergone RANKL-induced differentiation and maturation on bovine bone slices; we found that 50 and 100 nM Elt treatment for 48 h significantly reduced the area percentage of resorption lacuna formed by osteoclasts per unit area of bone slices (Figures 3D–F). These results suggested that Elt can not only inhibit the formation of the “sealing zone” of F-actin belts but also downregulate the bone-resorption activity of mature osteoclasts.

### Elt Inhibits Expression of Characteristic Genes and Proteins of Osteoclasts

**FIGURE 4:**
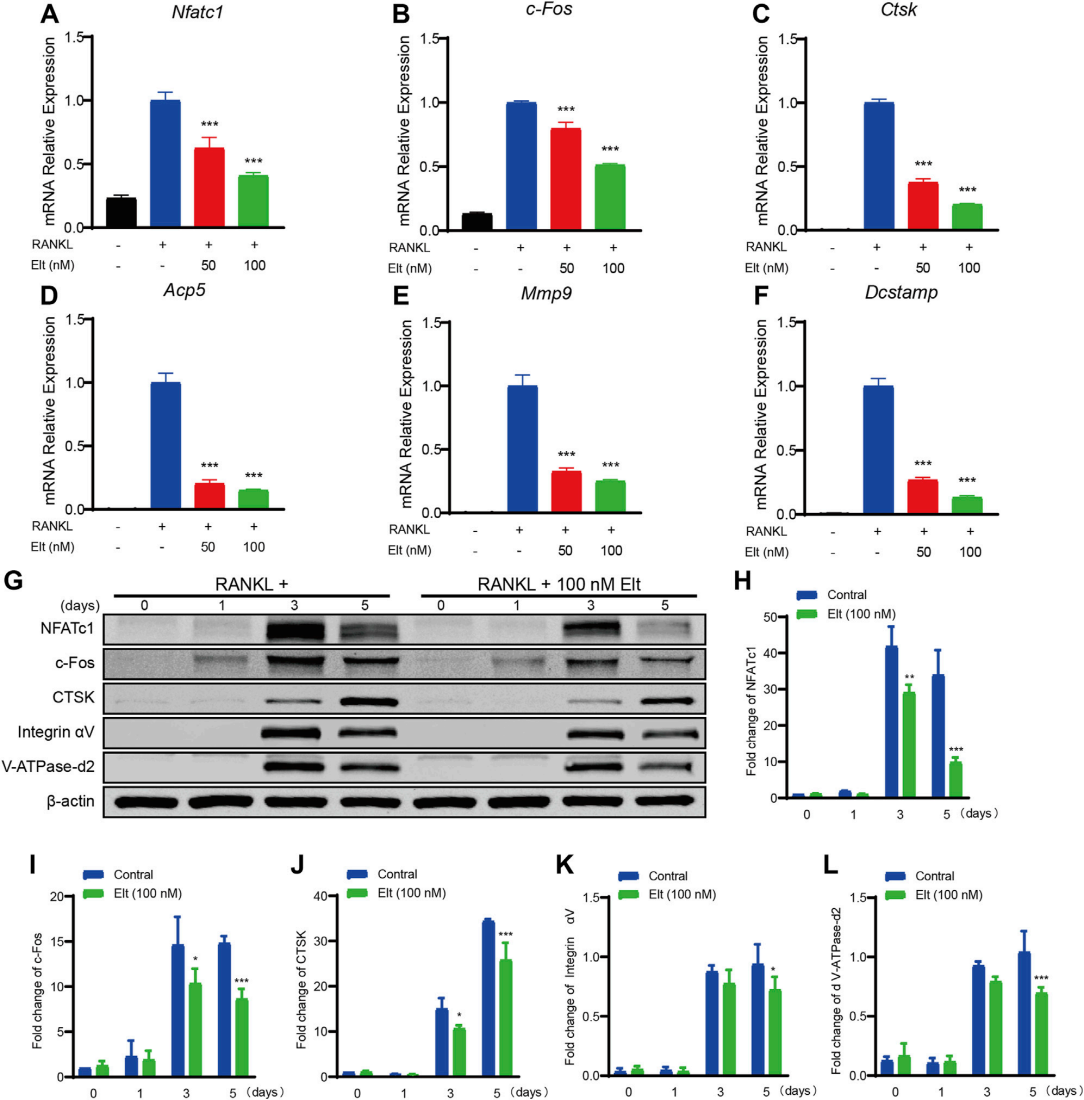
Elt inhibits expression of characteristic genes and proteins of osteoclasts. **(A–F)** The expression levels of the osteoclast-related specific genes *Nfatc1, c-Fos, Ctsk, Acp5, Mmp9 and Dcstamp* were analyzed by qPCR. **(G)** BMMs were stimulated with RANKL, with or without 100 nM Elt for 0, 1, 3, and 5 days, the expression of characteristic proteins was tested by western blots. **(H–L)** The expression of all the proteins mentioned above was standardized to β-actin expression. The above data are expressed as the mean ± SD; *n* = 3; *p < 0.05 relative to RANKL-induced controls.

Considering the aforementioned inhibitory effect of Elt on osteoclast formation and function, we next performed real-time PCR to analyze how Elt treatment affects the expression of genes related to RANKL-induced osteoclast formation and function. As compared with the expression in the control group stimulated with RANKL, Elt treatment significantly downregulated—in a dose-dependent manner—the expression of osteoclastogenesis-related genes (encoding NFATc1, c-Fos, and Dcstamp) as well as osteoclast function-related genes [encoding CTSK, Acp5, and matrix metalloproteinase (MMP)-9] (Table 1; Figures 4A–F). Moreover, we examined the expression of osteoclast characteristic proteins (NFATc1, c-Fos, and CTSK): In accord with the real-time PCR results, NFATc1 protein expression in osteoclasts decreased starting from the third day of Elt (100 nM) treatment, and the changes in the expression of c-Fos and CTSK also followed the same trend (Figures 4G–L). These findings suggest that Elt can block the expression of osteoclast-specific genes at both the transcriptional and translational levels, reducing osteoclast development and activity.

### Elt Does Not Markedly Affect the Initiation of NF-κB and MAPK Signaling Pathways by RANKL

**FIGURE 5:**
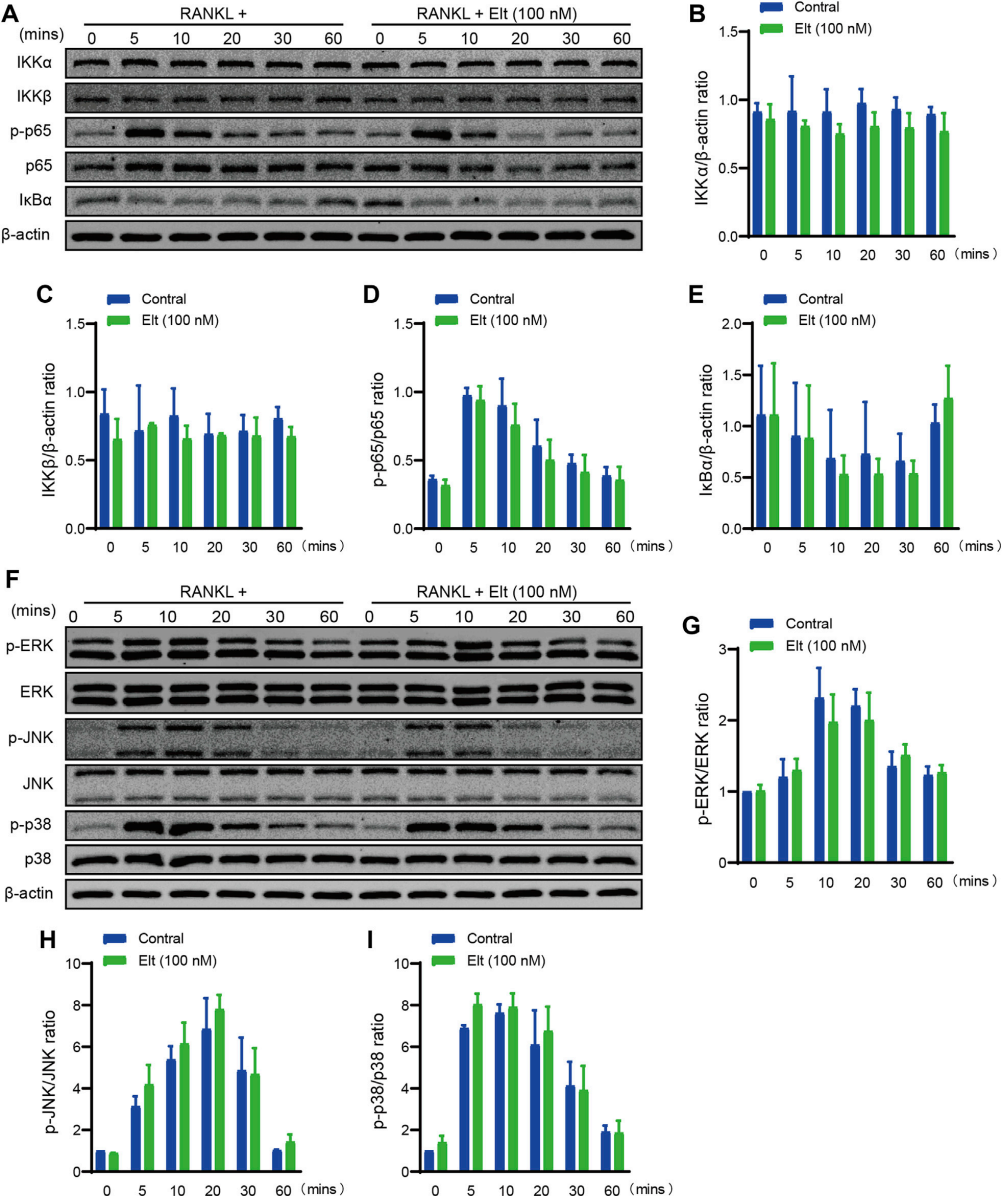
Elt does not markedly affect the initiation of NF-κB and MAPK signaling pathways by RANKL. **(A)** Representative Western Blot images of the effects of Elt on NF-κB pathways induced by RANKL within 60 min. **(B–E)** The quantitative analysis of IKKα, IKKβ, IκBα and p-p65 expression between the Elt-treated group and control group. **(F)** Representative Western Blot images of the effects of Elt on MAPK pathways induced by RANKL. **(G–I)** Quantification of the ratio of phosphorylated ERK, JNK and p38 to the corresponding total protein band. The above data are expressed as the mean ± SD; *n* = 3; Significant differences between the treatment and control groups are indicated as *p < 0.05.

By binding to the receptor RANK on osteoclast precursors and stimulating intracellular TRAF6 signaling, the activation of the NF-κB and MAPK signaling pathways, which are important in the creation and differentiation of osteoclasts, is induced by RANKL (Liu et al., 2020). Initiation of the NF-κB signaling cascade depends on IKK activation and IKK-mediated phosphorylation of IκBα. The results of western blotting here showed that Elt treatment did not significantly influence RANKL-induced IKK activation and p65 phosphorylation (Figures 5A–E). Moreover, we used western blotting to analyze the levels of phosphorylated p38, JNK, and ERK (p-p38, p-JNK, and p-ERK) in osteoclast precursors at 0–60 min after RANKL stimulation: Elt treatment also did not significantly alter the cellular content of p-p38, p-JNK, and p-ERK (Figures 5F–I). These findings indicate that Elt treatment has no effect on RANKL-induced initiation of NF-κB and MAPK signaling pathways activation.

### Elt Promotes Nuclear Accumulation of IκBα–NF-κB p65 and Withholds the NF-κB Signaling Pathway

**FIGURE 6:**
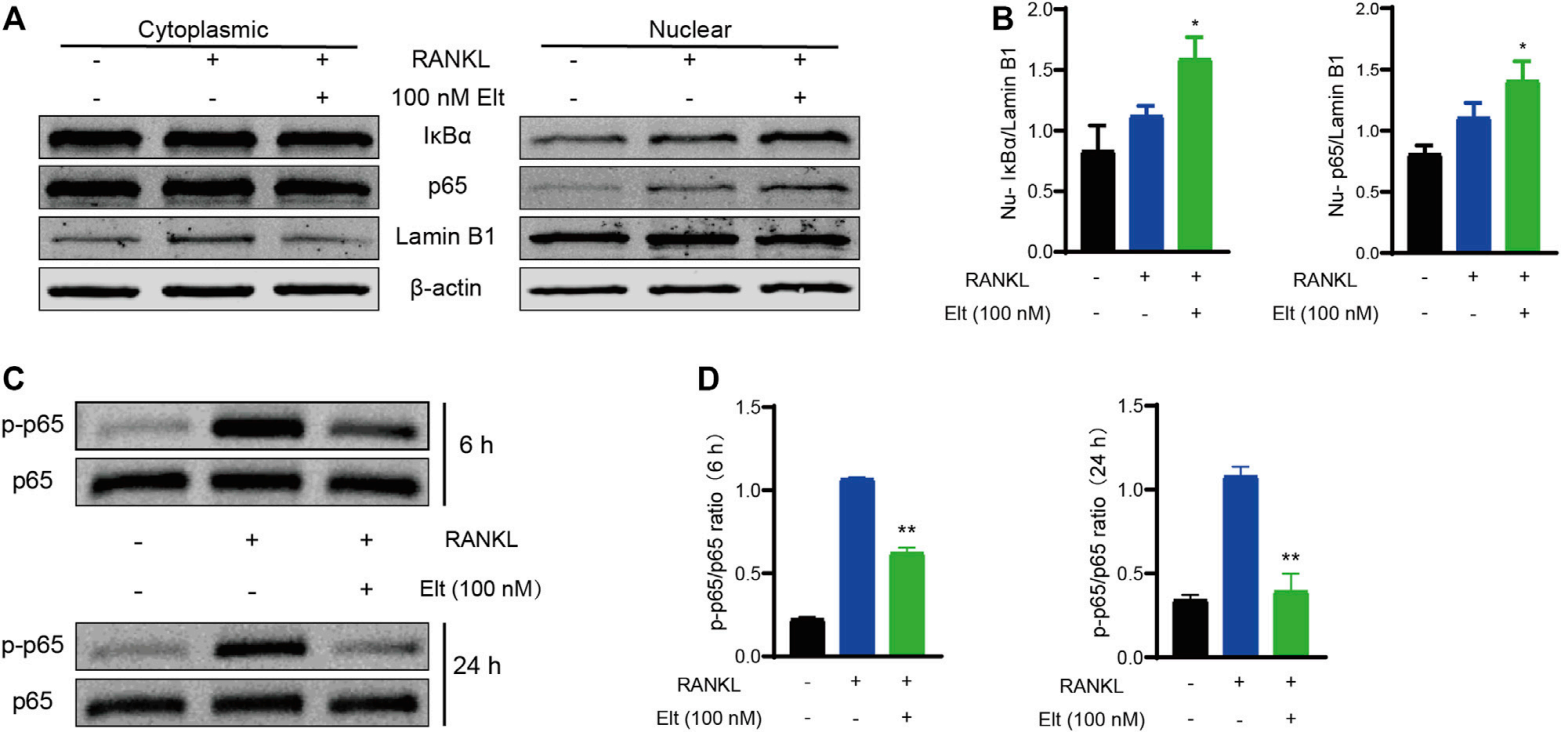
Elt promotes nuclear accumulation of IκBα–NF-κB p65 and withholds the NF-κB signaling pathway. **(A)** Cellular fractionation of BMMs cells shows similar increased nuclear levels of IκBα and NF-κB p65 upon Elt treatment in the presence of RANKL. Lamin B1 was used as nuclear protein marker; β-actin as a cytosolic protein marker. **(B)** Quantitative analysis of nuclear expression of IκBα and p65 in Elt treatment group. **(C)** Phosphorylation levels of p65 were detected within a short period of time after 6 h or 24 h of Elt treatment. **(D)** Quantification of the ratio of phosphorylated p65 to the corresponding total protein band. The above data are expressed as the mean ± SD; *n* = 3; Significant differences between the treatment and control groups are indicated as *p < 0.05.

**FIGURE 7:**
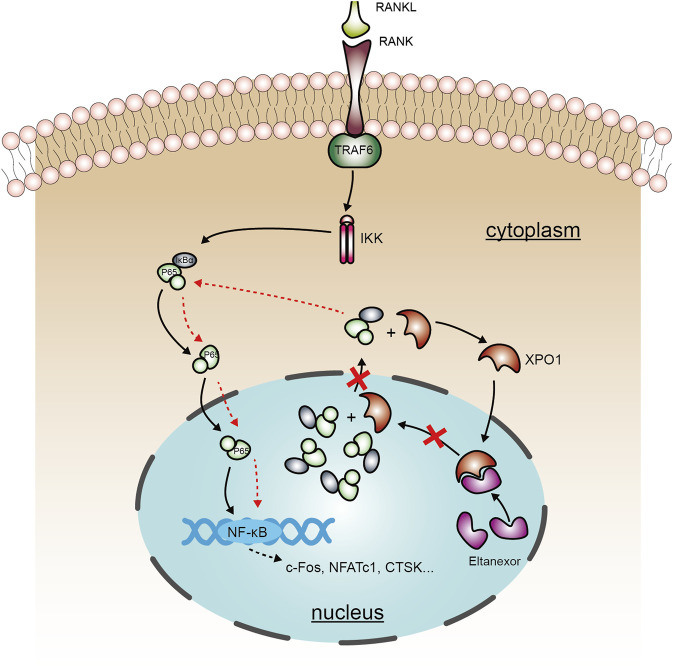
A schematic diagram showing the working model of the role of Elt in inhibiting osteoclastogenesis. Elt inhibits the NF-κB signaling pathway activated by RANKL in osteoclasts by blocking XPO1-dependent nucleus-to-cytoplasm transfer of NF-κB, and this effect strengthens the mechanism of inhibiting excessive activation of osteoclasts.

The results of the aforementioned experiments showed that Elt does not markedly affect the initiation of the NF-κB signaling pathway. Elt is reported to selectively inhibit nuclear export by targeting XPO1/CRM1, which can prevent the IκBα–NF-κB p65 complex from exiting the nucleus (Boons et al., 2021). Therefore, we separated the nucleus from the cytoplasm and detected the levels of IκBα and p65 in the two subcellular compartments: After Elt treatment, the nuclear content of IκBα and p65 was significantly increased (Figures 6A,B), whereas the levels of the proteins in the cytoplasm were not altered significantly. However, the overall cellular level of phosphorylated p65 (p-p65) was significantly decreased (Figures 6C,D). These experimental results indicate that Elt treatment can prevent the continuous activation of the NF-κB signaling pathway by retaining IκBα-p65 in the nucleus (Figure 7).

### Elt can Attenuate Bone Loss Caused by Ovariectomy

**FIGURE 8:**
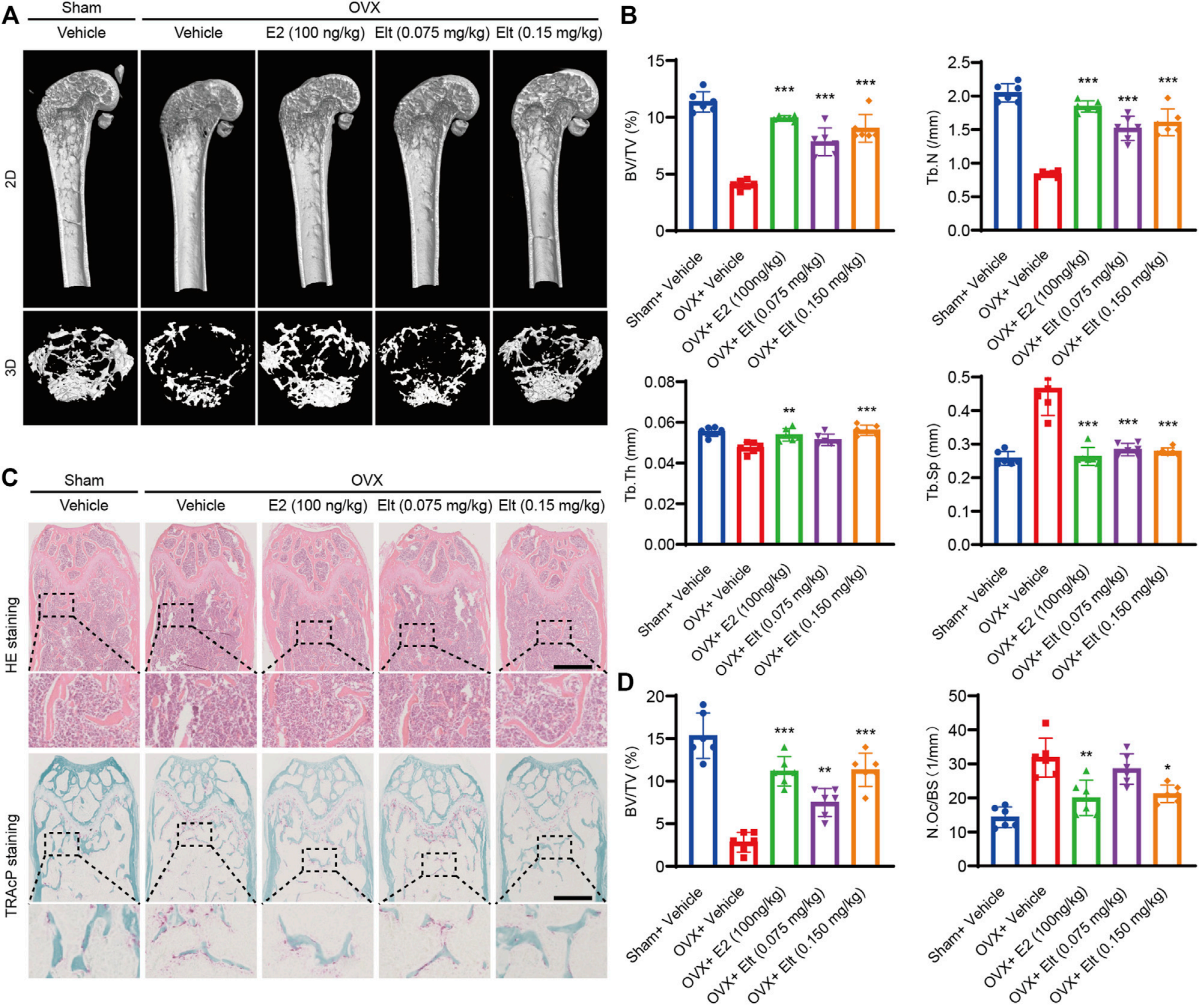
Elt attenuates bone loss caused by ovariectomy. **(A)** Femur structures in each group were captured by high resolution μCT and postprocessed by 2D or 3D computer reconstruction. **(B)** Quantitative analyses of parameters regarding bone microstructure. **(C)** Representative photographs of HE and TRAcP staining from each treatment group (scale = 500 μM). **(D)** Quantitative analysis of BV/TV of HE and the number of TRAcP^+^ osteoclasts in each group. The above data are expressed as the mean ± SD; *n* = 6; Significant differences between the treatment and control groups are indicated as *p < 0.05.

Lastly, to verify the effect of Elt on osteoclast production and function in the *in vivo* environment, we established a mouse osteoporosis model (the OVX mouse) by performing ovariectomy and then used micro-CT and histopathological methods to analyze bone volume and microscopic morphology of the mouse femur. After vehicle treatment, the femoral bone mass of the mice in the OVX group was significantly decreased, the bone volume/total volume ratio was significantly diminished, the number of bone trabeculae was decreased, and the density of connective tissue was increased. By contrast, the bone mass of the mice in the Elt group and the estrogen (E2) group was significantly increased, which suggests that Elt and estrogen therapy effectively reduced bone loss in the osteoporosis model mice (Figures 8A,B). Moreover, the same trend in terms of the inhibition of bone loss was revealed in the microhistological analysis of decalcified distal femur sections performed using H&E and TRAcP staining (Figures 8C,D). Conversely, liver and kidney morphology and texture of the mice in the Elt-treatment group were not significantly different from those in the other groups (Supplementary Figure S1). These results indicate not only that Elt can inhibit osteoclast activity *in vitro*, but also that Elt can be used to reduce excessive bone resorption caused by estrogen deficiency *in vivo*. Figures are compiled from each group’s typical pictures.

## Discussion

The process of bone metabolism requires the participation of diverse cells, among which osteoblasts and osteoclasts are the basic elements (Saylor et al., 2011). Osteoclasts are key bone metabolism cells that play a role in bone development, bone resorption, and bone-mass regulation. When osteoclasts are abnormally activated, the cells cause changes in the bone microenvironment, which is then followed by a series of bone-related diseases, such as osteoporosis and rheumatoid arthritis (Minoshima et al., 2019). Thus, considerable research interest is devoted to using the mechanism of osteoclast differentiation and bone resorption to identify suitable targets for the treatment of patients with such bone diseases. However, previously employed hormone therapy, therapies involving phosphate or calcitonin, and other methods for treating bone loss have produced several adverse reactions, such as cardiovascular events, gastrointestinal intolerance, and osteonecrosis of the jaw (Zhang et al., 2014; Sakaki et al., 2018; Sonoda et al., 2020). Moreover, although increasing numbers of compounds, including naturally occurring compounds, plant extracts, and small-molecule inhibitors, have been reported to exert various pharmacological effects on osteoclast differentiation and function (Cheng et al., 2012; Jin et al., 2019), these studies have focused mainly on the molecular biological mechanisms of action of the compounds. Therefore, urgent demand still exists for identifying innovative methods to inhibit overactivated osteoclasts as well as exploring the molecular biological mechanisms of the inhibited activity. In this study, we demonstrated that Elt inhibited osteoclast production and function by trapping IκBα and p65 in the nucleus and thereby inhibiting NF-κB activity and effectively reversing bone loss in ovariectomized mice. This cellular sorting of a critical protein complex represents a newly identified mechanism for targeting in the drug treatment of osteoporosis.

Proper cellular localization of proteins is crucial for the physiological activities of cells. In eukaryotes, a membrane system divides cells into the cytoplasm and nucleus (Akopian et al., 2013). Whereas the nucleus is the site where the genomic material is stored and gene expression is regulated to modulate cellular functions, the cytoplasm is the compartment where, after gene transcription, the transcripts are translated and modified to generate proteins; the cytoplasm is also the main site of cellular metabolism. The normal functioning of intracellular regulatory proteins requires appropriate temporal and spatial localization of the proteins (Martin and Jans, 2021). For example, tumor-suppressor proteins perform their functions when the proteins are translated and modified in the cytoplasm and then transported to the nucleus. Conversely, mislocalization or abnormal regulation of proteins leads to diverse diseases, including inflammation, cancer, and autoimmune diseases (Liu and Hu, 2016). Aberrant protein localization can be caused by numerous factors, such as protein misfolding, abnormal posttranscriptional modification of proteins, changes in the protein signal sequence, and abnormal protein nuclear transport, and particularly notable here among these factors is the nuclear and cytoplasmic mechanism that regulates the nuclear positioning of key proteins (Shin et al., 2013); this is because the nuclear and cytoplasmic transport of macromolecules requires active transport mediated by nuclear transport proteins such as XPO1.

The nuclear-export protein XPO1, which is also called exportin-1 and CRM1, was discovered in 1997 and is the most critical exporter of nuclear transport protein β (karyopherin-β/importinβ) family members, which are one of the nuclear transport receptors that act mainly by recognizing the NLS and nuclear-export signal of proteins to participate in the nuclear and cytoplasmic transport of intracellular materials (Mukherjee et al., 2015; Kashyap et al., 2021). XPO1 is capable of mediating the nuclear export of over 200 proteins, including tumor suppressor and growth-regulator proteins such as p21, FOXO1, p53, and NF-κB. XPO1 has emerged as a possible anticancer treatment target due to its high expression in tumor cells (Liao et al., 2021). Inhibition of XPO1-mediated nuclear transport can prevent the excessive nuclear export of tumor-suppressor proteins, antiapoptotic proteins, and growth-regulator proteins and thereby maintain the effective concentration of such proteins in the nucleus and exert a tumor-suppressor effect (Currier et al., 2019).

Leptomycin B (LMB), the first natural inhibitor of XPO1 to be discovered, was reported to bind irreversibly to Cys528 in XPO1 and inhibit the formation of a nuclear-transport ternary complex (Crochiere et al., 2015). Subsequent preclinical studies showed that LMB exerted antitumor effects in various mouse models generated using tumor cells, but the dose-limiting toxicity of LMB led to clinical trials being interrupted (Cela et al., 2020). Karyopharm Therapeutics, a US pharmaceutical company, has created a family of SINE chemicals, or small-molecule nuclear export inhibitors, including KPT-185, KPT-276, verdinexor (KPT-335), Sel (KPT-330), and Elt (KPT-8602). Sel is an orally administered, selective nuclear-export inhibitor that has been used in clinical phase I and phase II studies of hematological tumors, osteosarcoma, and other solid malignant tumors (Muz et al., 2017; Liao et al., 2021). However, because 60% of the patients exhibited serious adverse reactions and systemic toxicity, Sel was rejected by the US FDA for approval for marketing. Elt is a second-generation oral XPO1 inhibitor developed by Karyopharm Therapeutics. Relative to the first-generation XPO1 inhibitor Sel, Elt has shown similar *in vitro* efficacy in preclinical models and furthermore, offers the advantages of a broad therapeutic spectrum, low blood-brain-barrier penetration, and strong tolerance (Vercruysse et al., 2017; Wang et al., 2018). Although Elt is currently being tested in fewer preclinical and clinical trials than Sel, the broader therapeutic spectrum and lower central nervous system permeability of Elt led to relatively higher safety and tolerability, and this is expected to alleviate the adverse reactions currently observed with XPO1 inhibitors (Hing et al., 2016; Etchin et al., 2017).

Excessive activation of the NF-κB signaling pathway is a major causative factor of inflammatory diseases, immune diseases, and cancer, and this overactivation is also a key factor in osteoclast hyperfunction (Xu et al., 2009). IκBα, a negative regulator of the NF-κB signaling pathway, is synthesized in the cytoplasm and transferred to the nucleus, where the protein competes with DNA to bind to NF-κB; subsequently, with the assistance of XPO1, the NF-κB/IκBα complex is transported to the cytoplasm, and consequently, NF-κB signaling is terminated (Lang and Jou, 2021). When stimulation by external signals causes the dissociation of the NF-κB/IκBα complex, NF-κB is released and its downstream signaling pathway is activated. Therefore, using XPO1 inhibitors to block NF-κB shuttling between the nucleus and the cytoplasm, allowing inactive NF-κB to accumulate in the nucleus, can inhibit the continuous activation of the NF-κB signaling pathway. Notably, Elt effectively inhibits the nuclear export of the NF-κB/IκBα complex, and this inhibitory effect is markedly reduced in XPO1-knockout cells (Zhang et al., 2013). Here, we confirmed that Elt exerts no cytotoxic effect on osteoclasts within the effective concentration range of the drug, and further that Elt potently inhibits the formation and function of osteoclasts.

The RANKL signaling pathway holds considerable physiological significance in relation to osteoclast formation (Sousa et al., 2016). Following activation by RANKL, the receptor RANK recruits the adaptor protein TRAF6 and mediates TRAF6 signaling by rapidly triggering a series of downstream signaling events, with the NF-κB and MAPK signaling pathways being the two early pathways that respond to RANKL stimulation. The activation of the NF-κB and MAPK signals increases the expression of the transcription factors c-Fos and NFATc1 (Wang et al., 2017). The activator protein-1 transcription factor complex contains c-Fos, which collaborates with NFATc1 to stimulate the expression of downstream osteoclast-specific proteins, including TRAcP, CTSK, Dcstamp, and MMP-9 (Phromnoi et al., 2021). We found that Elt can inhibit the transcriptional activity of NFATc1 and c-Fos and downregulate the expression of genes related to osteoclast formation and function. We speculated that this inhibitory effect might be related to the blocking of the NF-κB and MAPK signaling pathways by Elt. Considering this possibility, we measured the degradation of IκBα and the phosphorylation of p65, JNK, p38, and ERK within a short treatment period (1 h), which revealed that Elt exerted no marked effect on the initiation of NF-κB and MAPK signaling pathways. Because Elt-induced protein accumulation in the nucleus is expected to occur over a relatively longer timeframe (Tajiri et al., 2016), we next extended the detection time to 24 h; our results showed that Elt strongly inhibited p65 phosphorylation, although this inhibitory effect was unrelated to the phosphorylation level of IKK and was due to Elt blocking NF-κB exit from the nucleus. This finding can partly explain the mechanism by which the RANKL-induced osteoclast differentiation-related transcription factors c-Fos and NFATc1 are downregulated by Elt. Our findings show that Elt promotes the nuclear accumulation of NF-κB/IκBα by inhibiting XPO1 in a concentration-dependent manner and thus blocks RANKL-activated NF-κB nuclear-cytoplasmic shuttling to attenuate the osteoclast differentiation and bone-resorption activity regulated by NF-κB signaling.

Measurement here of the strong inhibitory effect of Elt at the cellular level *in vitro* led us to construct the OVX mouse model to evaluate the therapeutic potential of Elt in alleviating bone loss *in vivo* and the potential metabolic toxicity of the drug. The results of micro-CT analysis showed that in OVX mice, bone mass was decreased and the trabecular bone was markedly reduced, which suggested that the osteoporosis model was successfully established. Notably, the mice in the Elt-treatment group had an increase in the number of bone trabeculae and a decrease in trabecular separation when compared to the control OVX animals, and the BV/TV index of the Elt-treated mice was considerably greater than that of the control OVX mice. Histomorphological research revealed that Elt therapy considerably increased the bone mass of OVX mice but had no cell toxicity damage in the major bodily organs. These results raise the possibility that Elt can be applied in the prevention and treatment of osteoporosis.

In summary, based on our findings *in vitro*, we further explored the mechanism by which Elt inhibits osteoclasts. We found that Elt inhibits the NF-κB signaling pathway activated by RANKL in osteoclasts by blocking XPO1-dependent nucleus-to-cytoplasm transfer of IκBα–NF-κB p65, and this effect strengthens the mechanism of inhibiting excessive activation of osteoclasts. Importantly, the effect of Elt in the treatment of osteoporosis has also been confirmed *in vivo*. Elt-induced nuclear sequestration of inactive IκBα–NF-κB p65 complexes blocks NF-κB activation by extracellular stimuli, a specific mechanism that has not been reported in studies of targeted inhibition of osteoclasts. Our study not only enriches the understanding of the subcellular localization of the IκBα–NF-κB p65 complex on NF-κB regulation of osteoclast physiological functions but also provides a new way to attenuate NF-κB activity in osteoclast. Especially, for the targeted inhibition of osteoclasts, new drugs are emerging one after another, and our research not only provides a potential drug, but also provides a new molecular mechanism, and this new mechanism may provide more drug options for osteoclast inhibition. Notably, we only analyzed the XPO1-dependent nuclear-cytoplasmic transfer and localization of a single protein, but XPO1 can mediate the nucleus-to-cytoplasm transport of more than 200 proteins (Wettersten et al., 2014). This requires us not only to further explore other potential mechanisms of Elt regulating osteoclasts, but also to explore the important role of nuclear-cytoplasmic transport mechanisms in osteoclasts, which we will continue to explore in future work. Thus, using small-molecule compounds to regulate XPO1-mediated nuclear-cytoplasmic shuttling of specific proteins and thereby restoring the normal distribution and function of critical proteins in the nucleus could represent a promising therapy method for associated illnesses.

## Data Availability

The original contributions presented in the study are included in the article/Supplementary Material, further inquiries can be directed to the corresponding authors.

## Supplementary Material

Supplementary Figure S1 Elt has no obvious toxic effect on the liver or kidney in ovariectomized mice. Representative images of hematoxylin-eosin staining of the liver and kidney; *n =* 3*.*

## Glossary

BMM: bone marrow-derived macrophage
BV/TV: bone volume/tissue volume
CCK-8: cell counting kit-8
CRM1: chromosome region maintenance 1
CTSK: cathepsin K
DMSO: dimethyl sulfoxide
Elt: eltanexor
ERK: extracellular signal-regulated kinase
FBS: fetal bovine serum
GAPDH: glyceraldehyde 3-phosphate dehydrogenase
H&E: hematoxylin and eosin
IKK: IκB kinase
IκB: nuclear factor-κB inhibitor
JNK: c-Jun N-terminal kinase
LMB: leptomycin B
MAPK: mitogen-activated protein kinase
M-CSF: macrophage colony-stimulating factor
MEM: minimal essential medium
MMP: matrix metalloproteinase
N.Oc/BS: number of osteoclast/bone surface
NFATc1: nuclear factor of activated T cells 1
NF-κB: nuclear factor-κB
NLS: nuclear localization signal
OVX mouse: ovariectomized mouse
P/S: penicillin/streptomycin
PBS: phosphate-buffered saline
PFA: paraformaldehyde
PI3K/AKT: phosphatidylinositol 3-kinase/protein kinase B
qPCR: quantitative PCR
RANK: receptor activator of NF-κB
RANKL: RANK ligand
RFP: red fluorescent protein
Sel: selinexor
SPF: specific pathogen-free
Tb.N: number of trabeculae
Tb.Sp: trabecular separation
Tb.Th: trabecular thickness
TNF: tumor necrosis factor
TRAcP: tartrate-resistant acid phosphatase
TRAF6: TNF receptor-associated factor 6
XPO1: exportin-1
