# Supplementary figures and images for: Anti-Osteoclast Effect of Exportin-1 Inhibitor Eltanexor on Osteoporosis Depends on Nuclear Accumulation of IκBα–NF-κB p65 Complex
Source: Front Pharmacol. 2022 Aug 30;13:896108. doi: 10.3389/fphar.2022.896108 (PMC9468713; doi:10.3389/fphar.2022.896108)

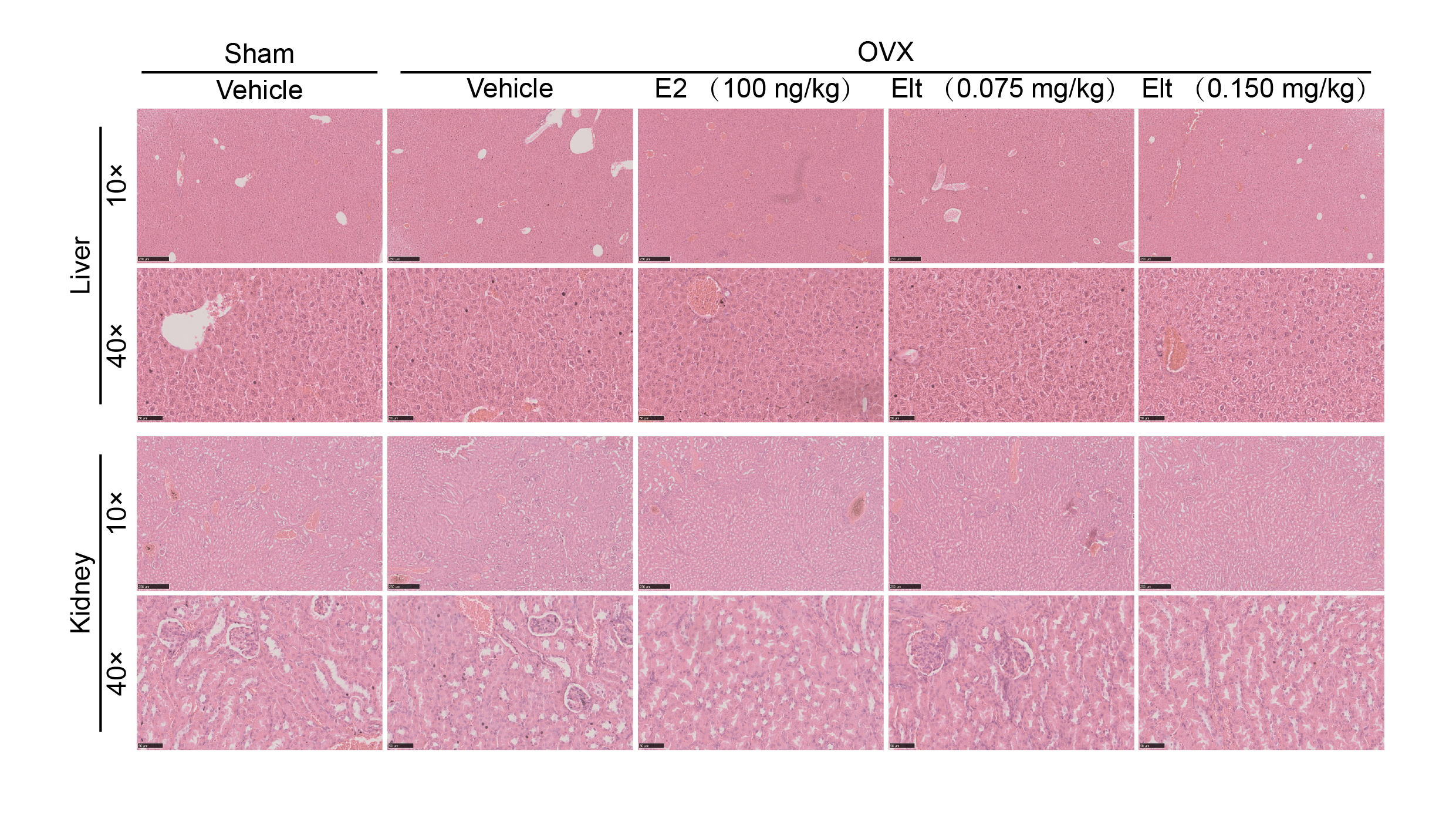

Supplement: Supplementary file 1 [file Image1.tif]
